# Trend of Antenatal Depression and Its Risk Factors Among Pregnant Women in China From 2016 to 2021: A Repeated Cross-Sectional Study Under Multiple Fertility Policy Adjustments and Economic Development

**DOI:** 10.1155/da/6823160

**Published:** 2025-04-15

**Authors:** Chi Zhou, Fang Tan, Xu Li, Jingchun Chen, Qunfang Huang, Xiaoling Lin, Falin Zhao, Qi Yuan

**Affiliations:** ^1^School of Public Administration, Hangzhou Normal University, Hangzhou 311121, Zhejiang, China; ^2^School of Medicine and Health Management, Tongji Medical College, Huazhong University of Science and Technology, Wuhan 430030, Hubei, China; ^3^School of Health Medicine, Guangzhou Huashang College, Guangzhou 511300, Guangdong, China; ^4^Research Division, Institute of Mental Health, Singapore 539747, Singapore

**Keywords:** antenatal depression, Chinese pregnant women, fertility policy, repeated cross-sectional

## Abstract

**Background:** The objective of the current study was to evaluate the trend and risk factors of antenatal depression (AD) among Chinese women in the third trimester of pregnancy, taking into account the impact of multiple fertility policy adjustments and economic development in China.

**Method:** A repeated cross-sectional study design was used. A total of 3404 pregnant women at 30–42 weeks' gestation were recruited from the two largest maternity hospitals in Zhejiang Province, China, between 2016 and 2021.

**Results:** The prevalence of AD among women in their third trimester of pregnancy had significantly increased from 31.8% to 60.6% (*p*  < 0.001) from 2016 to 2021. Pregnant women aged between 18 and 24 years reported the highest prevalence of AD. Those aged between 25 and 34 years (odds ratio [OR], 0.788; 95% confidence interval [CI]: 0.630–0.985), with a better self-reported health status (OR, 0.929; 95% CI: 0.922–0.936) and higher perceived social support (OR, 0.948; 95% CI: 0.940–0.955), reported a lower prevalence of AD. Pregnant women who were housewives (OR, 1.399; 95% CI: 1.078–1.817), had an introverted personality (OR, 1.324; 95% CI: 1.119–1.568), and had experienced an unplanned pregnancy (OR, 1.303; 95% CI: 1.098–1.547) reported a higher prevalence of AD.

**Conclusions:** The significant increase in the prevalence of AD from 2016 to 2021 has caused concern in society. To improve the aforementioned situation, it is imperative to implement further initiatives to address the challenges faced by pregnant women, especially those who are housewives, have introverted personalities, and have experienced unplanned pregnancies.

## 1. Introduction

Antenatal depression (AD) is one of the most common mental disorders worldwide. The typical symptoms of AD include low mood, sleep disturbance, and somatic complaints [1, 2]. It is associated with a number of adverse fetal, obstetric, and neonatal outcomes, ranging from intrauterine growth restriction and low birth weight, to preterm birth [3]. About 20 years ago, the prevalence of AD was about 7% to 20% in high-income countries and 15% to 34% in low- and middle-income countries [3–5]. However, recent studies have shown that the prevalence of AD has increased to ~10% to 30% in high-income countries and 15% to 65% in low- and middle-income countries [6–8].

Over the past decade, China's fertility policy has undergone several major adjustments. Specifically, the universal two-child policy was implemented in October 2015, ending the long-standing one-child policy [9]. In June 2018, the three-child policy was introduced for families that met certain conditions (e.g., one-child couples and remarried couples), and it was then extended to all families in May 2021 [10]. Meanwhile, China has also experienced huge economic growth; for example, the gross domestic product (GDP) had increased from RMB 67,671 billion (about USD 9540 billion) in 2015 to RMB 110,000 billion (about USD 15,507 billion) in 2021. All of these could lead to changes in the prevalence of AD. From this point of view, it is worthwhile to study the trend of AD prevalence among pregnant women in China, which will provide valuable insights for the future adjustments of maternity policies and service planning in China.

Previous studies on AD have suggested that its risk factors may include low educational attainment and low income, economic inactivity, unplanned pregnancy, inadequate social support, poor physical health, and pregnancy-related complications [11, 12]. However, when examining the specific risk factors of AD in different countries, notable differences can also be observed. For example, marital dissatisfaction is associated with an increased risk of AD in Turkey [13], while in the United States, pregnant women from ethnic minorities showed more depressive symptoms [14]. In this case, risk factors associated with AD in pregnant women in China may also be different. This is another important research gap.

The aim of this study is to report the overall and subgroup trends of AD among Chinese women in the third trimester of pregnancy (30–42 weeks) from 2016 to 2021. It also aims to explore the risk factors for AD specific to pregnant women in China.

## 2. Method

### 2.1. Study Design and Data Collection

The data for this study were from a serial cross-sectional study designed to elucidate the temporal trend of AD among pregnant women in their third trimester of pregnancy in China between 2016 and 2021. Data were collected from the two largest maternity hospitals in Zhejiang Province, China (i.e., Women's Hospital, School of Medicine, Zhejiang University, and Hangzhou Women's Hospital). Participants who met the following inclusion criteria were included: (1) 18 years old and above, (2) women in their 30–42 weeks of pregnancy, and (3) who have undergone prenatal examinations at the two aforementioned hospitals. Individuals with cognitive disabilities were excluded from the current study.

Eligible participants who agreed to participate in the study were required to complete a questionnaire while waiting for their prenatal checkups at the hospital. The questionnaire was created using an electronic platform called Wen Juan Xing, which is a professional survey platform in China. A QR code was generated to link to the survey, which could be accessed by scanning the code with a mobile phone. Informed consent was obtained from all the participants prior to their participation.

### 2.2. Assessment of AD

The Chinese version of the Edinburgh Postnatal Depression Scale (C-EPDS) was used to assess AD in the current study. It was translated and validated by Lee in 1998 [15]. This scale measures a range of symptoms including anhedonia, self-blame, anxiety, fear or panic, and self-harm ideas [16]. The scale consists of 10 items and each with four responses ranging from 0 (not at all) to 3 (always). The total score is calculated by summing the scores for each item, with a range of 0–30. In this study, the cutoff score for mild-and-moderate AD is 9–12, and for major AD, it is 13 and above (referring to the diagnosis criteria of the Diagnostic and Statistical Manual of Mental Disorder-IV) (Gonidakis et al., 2016). This scale has been used in several previous studies and showed good internal reliability (i.e., Cronbach' alpha 0.714–0.760), good test–retest reliability (i.e., 0.803–0.814), and good content validity ratio of 0.933 [17–19]. In the current study, the internal reliability of the C-EPDS was 0.883.

### 2.3. Sociodemographic and Health-Related Risk Factors

Sociodemographic characteristics were collected, including age, district (i.e., urban or rural), education level (i.e., high school or below or undergraduate college), employment status (i.e., employed or housewife), average personal monthly income (i.e., ≤RMB 4500 [approximately USD 634] or >RMB 4500), self-reported personality (i.e., introvert or extrovert), planned pregnancy (i.e., yes or no), and previous unsuccessful experience (e.g., abortion) (i.e., yes or no), husband's education (i.e., high school or below or undergraduate college), husband's occupation (i.e., white collar, blue collar, self-employed, and unemployed).

Participants' self-reported health status was measured using the visual analog scale (VAS) of the EuroQol Five Dimensions Questionnaire (EQ-5D). The EQ-5D scale is one of the instruments used to measure health-related quality of life. It consists of two parts: a five-item self-reported problems section and a VAS rating [20]. Self-reported health status was scored on a 20 cm vertical line where the best and worst imaginable health states were assigned a score of 100 and 0, respectively [20]. A higher score indicates a better level of health status. In the previous studies, this instrument showed good internal reliability (i.e., Cronbach' alpha was 0.700–0.743, and test–retest reliability was 0.850–0.990) [21, 22].

Social support was measured by the Chinese version of the Perceived Social Support Scale (PSSS). This scale was developed by Zimet and subsequently adapted by Jiang QJ [23, 24]. It has 12 items and measures three domains including social support from family members, from friends, and from significant others. Each item is scored on a scale from 1 (very strongly disagree) to 7 (very strongly agree). The total score ranges from 12 to 84, with higher scores indicating higher levels of social support. The cut-off points are 12–36 for lower levels, 37–60 for middle levels, and 61–84 for higher levels. This scale also has a good internal reliability of 0.88 and a good test–retest reliability of 0.85 [25]. In the current study, its internal reliability was 0. 94.

### 2.4. Statistical Analyses

Statistical analyses were performed via SPSS version 23.0 and R software (version 4.2.1). Descriptive analyses were performed for the sociodemographic variables, with the prevalence of AD presented according to these characteristics. The linear-by-linear association trend test was used to assess the temporal trend of AD prevalence over time. The variable “years” was treated as an ordinal variable, while the prevalence of AD was categorized into different time intervals. This analysis evaluates the significant linear trend in the prevalence of AD across these ordered time categories. Binary logistic analysis was used to explore the potential risk factors for AD. The full model used AD as the outcome, with “total scores <9′ as the reference group [26] and regressed on sociodemographic and health-related risk factors. The significance level for all regressions was set at 0.05. The measure of association was presented as odds ratios (ORs) with 95% confidence intervals (CIs). Age was not standardized in the current study, as there is no standard age structure of pregnant women and fertility policy adjustments may affect the number of pregnant women in different age groups.

## 3. Results

### 3.1. Sociodemographic Characteristics

A total of 3717 women in their third trimester of pregnancy were invited to participate between 2016 and 2021. Of these, 3404 (91.58%) had completed the full assessment and were then included in the subsequent analysis (Table 1, Figure S1), with a mean age of 27.5 years old (standard deviation [SD] = 3.9). Approximately half of the subjects (55.6%) were from rural areas, and the majority of them (54.8%) had an education level of high school or below. The proportion of women who were housewives was 13.3%. About half of the subjects (52.8%) reported an average personal monthly income of more than RMB 4500. More than half of the subjects (58.3%) reported extroverted personality, while 57.6% of them had planned pregnancies. About one-third of the subjects had previous unsuccessful pregnancy experiences (37.8%). More than half of the subjects' husbands (56.1%) had attained a high school education or below, while 59.7% of their husbands were blue collar workers.

**Figure 1:**
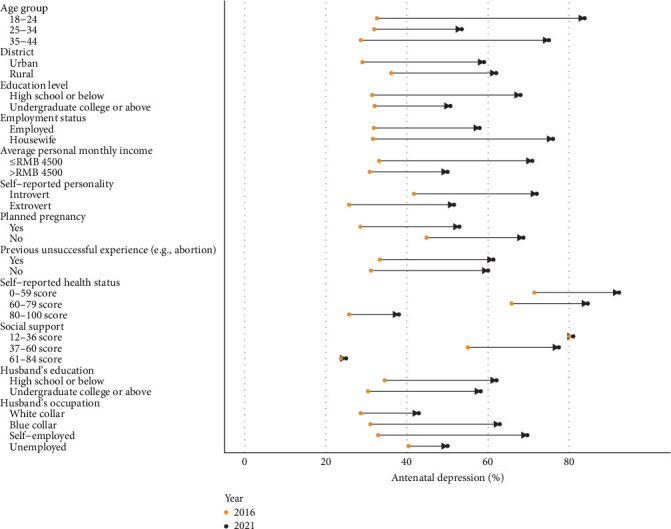
Changes in sociodemographic characteristics of pregnant women in the third trimester in China from 2016 to 2021.

Over the 6-year period, the proportion of pregnant women aged between 18 and 24 years increased from 8.0% to 21.8%, those aged between 25 and 34 years decreased from 88.3% to 75.9%, and those aged between 35 and 44 years decreased from 3.7% to 2.4%. The proportion of pregnant women from rural areas increased from 39.7% to 54.1%, while those with a low education level increased from 33.4% to 57.1%. The proportion of pregnant women who were housewives increased from 6.6% to 14.7%, while those with an average monthly income below RMB 4500 increased from 44.4% to 50.6%. In addition, the proportion of pregnant women with an introverted personality increased from 38.1% to 44.1%. The proportion of pregnant women who had undergone an unplanned pregnancy increased from 20.3% to 48.8%, and the proportion who had a previous unsuccessful pregnancy (e.g., abortion) increased from 30.9% to 44.1%. The proportion of pregnant women with self-reported health status scores between 80 and 100 decreased from 85.0% to 54.1%, while those with self-rated social support scores between 61 and 84 decreased from 74.7% to 32.9% (Figure 1).

### 3.2. Trends in AD Prevalence

**Figure 2:**
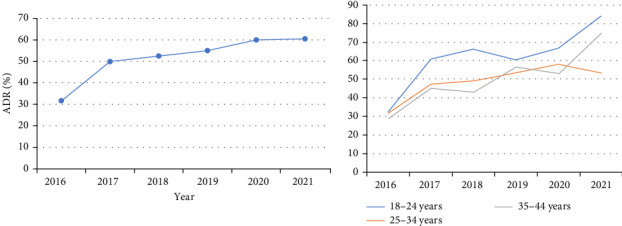
Trends in ADR from 2016 to 2021 year among third trimester pregnancy women.

**Figure 3:**
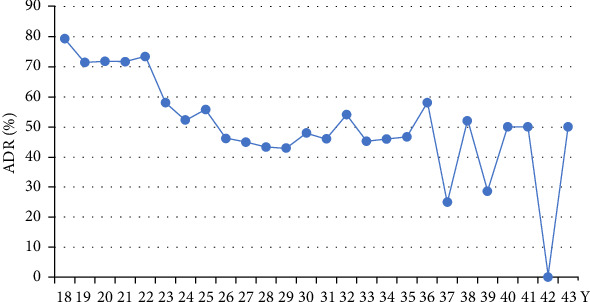
Trends in ADR among 18–43-year-old pregnancy women. ADR, antenatal depression rate.

The prevalence of AD increased significantly from 31.8% (95% CI: 28.0–35.6) in 2016 to 60.6% (95% CI: 53.2–68.0) in 2021 (time trend *p*  < 0.001), almost doubling over this period (Figure 2). Looking at the trends in AD prevalence in different age groups, the 18–24-year group showed a relatively large increase in AD prevalence, rising from 32.6% in 2016 to 83.8% in 2021. In contrast, the 25–34-year-old group showed a smaller increase, with AD prevalence rising from 31.9% in 2016 to 53.5% in 2021 (Figure 2). In terms of severity, the prevalence of mild-and-moderate AD increased slightly from 23.3% in 2016 to 27.6% in 2021. In contrast, the prevalence of severe AD showed a more pronounced increase, rising from 8.6% in 2016 to 32.9% in 2021 (Figure S2). In terms of age-specific prevalence, the highest prevalence of AD was reported among pregnant women aged 18–24 years, with 79.3% at 18 years old and 73.3% at 22 years old (Figure 3). In addition, the highest level of severe AD was reported among individuals aged 18–20 years (Figure S3).

### 3.3. Factors Influencing AD

Logistic regression analysis suggested that pregnant women aged between 25 and 34 years (OR, 0.788; 95% CI: 0.630–0.985), with a higher self-reported health status (OR, 0.929; 95% CI: 0.922–0.936) and greater perceived social support (OR, 0.948; 95% CI: 0.940–0.955), reported a lower prevalence of AD. In comparison, pregnant women who were housewives (OR, 1.399; 95% CI: 1.078–1.817), who had an introverted personality (OR, 1.324; 95% CI: 1.119–1.568), and who had experienced an unplanned pregnancy (OR, 1.303; 95% CI: 1.098–1.547) were more likely to report a higher prevalence of AD (Table 2).

## 4. Discussion

Using a repeated cross-sectional survey design, our study found a significant upward trend in the prevalence of AD from 2016 to 2021 among women at their third trimester of pregnancy in China, with a particularly notable increase in the prevalence of severe AD. Overall, the absolute value of the AD rate had increased by 30% over the past 6 years among our sample, compared to about 10% in other countries over the past decade [4, 5]. This change was significantly more pronounced than that observed in lower–middle-income countries [27] and therefore deserves greater attention. Previous studies have shown that AD is the strongest risk factor for postpartum depression (PD), with an estimated increase in the ratio of PD of 1.5–3.8. Furthermore, PD has been shown to have a negative impact on the intention to have a subsequent pregnancy [28–31]. This phenomenon can also be observed in our dataset—as the prevalence of AD increased, the number of pregnant women decreased. Thus, there is an urgent need to reduce the prevalence of AD, which may also help to address the current low fertility rate in China.

It is also notable that a number of other noteworthy changes have been observed. A preliminary analysis revealed an immediate increase in the number of pregnant women and a sharp rise in AD prevalence following the implementation of the two-child policy, the first round of adjustments. In addition, the demographic profile of pregnant women was found to have changed over the 6 years. For example, the proportion of pregnant women between the ages of 18 and 24 had changed significantly, increasing from 8% in 2016 to 21.8% in 2021. The proportion of individuals from rural areas, those with a low level of education, and those who were housewives had all increased. These phenomena seem to be the consequences of policy adjustments. The former may be attributed to the release of long-suppressed fertility demand, while the latter may be related to the easing of birth restrictions, which has increased the desire to have children among younger groups of people. However, these are only assumptions, and further studies, especially qualitative studies, are needed to explore this issue in greater depth and to identify the reasons behind the observed trend.

Our study identified some protective factors for AD, including older age, better self-reported health status, and higher perceived social support. These risk-protective factors are similar to those identified in the literature [32, 33, 50]. Firstly, our study suggests that pregnant women aged between 18 and 24 years have the highest prevalence of severe AD compared to those aged between 25 and 34 and between 35 and 44 years. This is particularly true for those aged between 18 and 20 years. Similar findings have been reported elsewhere, suggesting that younger maternal age increases the likelihood of depression during pregnancy [11, 34, 35]. There are several possible explanations in the Chinese context: first, the legal age of marriage for women in China is 20, so those who marry between the ages of 18 and 20, although a very small number, have to wait for a few years before they can obtain an official marriage certificate [36]. In contrast, pregnant women in an older age group would not face such challenges. Second, university students in China typically complete their undergraduate studies around the age of 22. However, if they become pregnant at this age, it would be very difficult for them to find or keep a decent job, and thus, they would face high financial burdens [37]. Pregnant women in an older age group, on the other hand, should have accumulated more financial savings and thus be less burdened in this regard. Third, self-reported health status was also identified as a protective factor for AD in our study. Previous studies among pregnant women have suggested that the presence of physical symptoms such as nausea, vomiting, and fatigue and present/past pregnancy complications are associated with a higher risk of AD, as these physical conditions would affect the daily lives of pregnant women [13, 38]. Both are typical health concerns for pregnant women. Finally, social support was found to be a protective factor for AD among pregnant women in China. It is a well-studied protective factor because social support may buffer the negative effects of stressors not only during the pregnancy period but also throughout life [39, 40].

We also identified some risk factors for AD among pregnant women in China, including being a housewife, having an introverted personality, and having an unplanned pregnancy. Being a housewife is associated with economic inactivity and is usually associated with financial strains that are stressful for pregnant women and their families [41]. Such financial burdens could be due to pregnancy and childbirth, and it could also be due to housing, education, and living [42]. To alleviate such financial burdens, it is suggested that more subsidies could be made available to them. The good news is that quite a number of cities have begun to provide subsidies for families with second and third children, suggesting that the government is working on this [43]. It has long been documented that personality plays an important role in depression in pregnant women [44]. Our study has further demonstrated the negative association between introversion and AD, suggesting that more attention needs to be paid to this subgroup. Finally, unplanned pregnancy can bring uncertainty and disruption. Such disruption can be at the individual level, leading to concerns such as whether the pregnancy will affect the career of the pregnant women; it can also be at the household level, leading to issues such as how to plan and organize subsequent caring tasks and responsibilities [45, 46]. All of these concerns can cause chaos and disruption in their current lives, resulting in increased stress. These are all valid concerns and challenges that faced by pregnant women, and more efforts are really needed, not only from the government, but also from the society.

There are several implications from the current study. Firstly, the low birth rate is a serious problem facing China today. Although the Chinese government has gradually relaxed the birth policy in recent years, the total number of pregnancies has actually decreased, except for the first round of adjustment from the one-child policy to the two-child policy, suggesting that adjusting the birth policy alone may not be a very good solution in this scenario. Instead, it is more important to understand the specific concerns and challenges faced by couples in different age groups in China and to develop solutions accordingly. This could be done through qualitative research and could be a direction for future research in this area. Secondly, apart from the high financial burden associated with babies, women in China may also face implicit discrimination from organizations and companies in terms of employment. Such discrimination could affect their decision to marry and become pregnant and ultimately affect the fertility rates. To address these issues, it is necessary to build a holistic fertility support system for them, which should include components such as economic support (e.g., maternity allowances), service support (e.g., childcare services), time support (e.g., extended parental leave), and legal support [43, 47]. This will require more effort from both society and government and may take a long time. Finally, the high prevalence of AD is also one of the main reasons for low fertility. To help pregnant women and their families to cope with AD, it is important to provide them with accessible mental health services that cover the entire perinatal period, and this could be something the government can work on.

The current study has several strengths. First, this is the first study to examine the prevalence of AD in China over a 6-year period using a repeated cross-sectional design. This design allowed us to observe changes in AD after fertility policy adjustments. The results are very informative for future policy planning. Second, the current study has a total sample size of 3404 which is quite large. This makes the findings on the relationship between AD and its risk factors more robust and reliable. The study also has some limitations. First, this study was conducted among pregnant women from Hangzhou, Zhejiang, China, which may affect the generalizability of the study findings. However, as one of the most developed cities in China, it is quite comparable to other developed cities such as Beijing, Shanghai, and Shenzhen. Second, although we used a repeated cross-sectional study design, it still suffers from the main disadvantage of the traditional cross-sectional design and cannot establish any causal relationships. In addition, the reproducibility of the results is limited due to the influence of the observation years and the implementation timeline of the fertility policies. Finally, it should be noted that the study did not collect data on personal lifestyle factors such as smoking, alcohol consumption, and physical activity nor did it collect data on pathological factors such as maternal diabetes, hypertension, central placenta previa, preterm births, and stillbirths. These factors may also influence the development of AD [11, 48, 49]. Instead, we only collected self-reported health information from the participants. While this information may provide some indication of the participants' health, it is not as detailed as the previously mentioned variables.

In conclusion, using a repeated cross-sectional study design, our study found that there was a significant upward trend in the prevalence of AD among women in the third trimester of pregnancy in China from 2016 to 2021. Although the Chinese government is gradually relaxing fertility restrictions, our results suggest that the total number of pregnancies is actually decreasing, except for the first round of adjustment from the one-child to the two-child policy, indicating that adjusting the fertility policy alone may not be a very good solution. The rapid increase of AD is an alarm to society. To help pregnant women in China and their families to cope with AD, it is important to provide them with accessible mental health services throughout the perinatal period, and this could be something the government can work on. Special attention should be given to pregnant women who are housewives, have introverted personalities, and have unplanned pregnancies. A holistic fertility support system that includes components such as economic support (e.g., maternity allowances), service support (e.g., childcare services), time support (e.g., extended parental leave), and legal support could be helpful.

## Figures and Tables

**Table 1:** Sample characteristics ($\overset{-}{X}$ ± SD/*n* [%]).

Variate	2016 (*n* = 572)		2017 (*n* = 993)		2018 (*n* = 772)		2019 (*n* = 526)		2020 (*n* = 371)		2021 (*n* = 170)		*Z* score^d^
	Total respondents (%)	EPDS scores ≥ 9(%)	Total respondents (%)	EPDS scores ≥ 9 (%)	Total respondents (%)	EPDS scores ≥ 9 (%)	Total respondents (%)	EPDS scores ≥ 9 (%)	Total respondents (%)	EPDS scores ≥ 9 (%)	Total respondents (%)	EPDS scores ≥ 9 (%)	
Age group													
18–24	46 (8.0)	15 (32.6)	207 (20.8)	126 (60.9)	186 (24.1)	123 (66.1)	131 (24.9)	79 (60.3)	102 (27.5)	68 (66.7)	37 (21.8)	31 (83.8)	12.6^a^
25–34	505 (88.3)	166 (31.9)	735 (74.0)	348 (47.3)	530 (68.7)	259 (48.9)	365 (69.4)	194 (53.2)	252 (67.9)	146 (57.9)	129 (75.9)	69 (53.5)	49.0^a^
35–44	21 (3.7)	6 (28.6)	51 (5.1)	23 (45.1)	56 (7.3)	24 (42.9)	30 (5.7)	17 (56.7)	17 (4.6)	9 (52.9)	4 (2.4)	3 (75.0)	4.2^c^
District													
Urban	345 (60.3)	100 (29.0)	442 (44.5)	186 (42.1)	297 (38.5)	138 (46.5)	207 (39.4)	114 (55.1)	142 (38.3)	95 (66.9)	78 (45.9)	46 (59.0)	72.3^a^
Rural	227 (39.7)	82 (36.1)	551 (55.5)	311 (56.4)	475 (61.5)	268 (56.4)	319 (60.6)	176 (55.2)	229 (61.7)	128 (55.9)	92 (54.1)	57 (62.0)	11.9^a^
Education level													
High school or below	191 (33.4)	60 (31.4)	532 (53.6)	309 (41.9)	481 (62.3)	284 (59.0)	332 (63.1)	203 (61.1)	234 (63.1)	141 (60.3)	97 (57.1)	66 (68.0)	28.0^a^
Undergraduate college or above	381 (66.6)	122 (32.0)	461 (46.4)	188 (40.8)	291 (37.7)	122 (41.9)	194 (36.9)	87 (44.8)	137 (36.9)	82 (59.9)	73 (42.9)	37 (50.7)	30.0^a^
Employment status													
Employed	534 (93.4)	170 (31.8)	864 (87.0)	406 (47.0)	646 (83.7)	323 (50.0)	447 (85.0)	234 (52.3)	314 (84.6)	184 (58.6)	145 (85.3)	84 (57.9)	64.1^a^
Housewife	38 (6.6)	12 (31.6)	129 (13.0)	91 (70.5)	126 (16.3)	83 (65.9)	79 (15.0)	56 (70.9)	57 (15.4)	39 (68.4)	25 (14.7)	19 (76.0)	6.5^c^
Average personal monthly income													
≤RMB 4500	254 (44.4)	84 (33.1)	459 (46.2)	268 (58.4)	375 (48.6)	222 (59.2)	259 (49.2)	159 (61.4)	173 (46.6)	102 (59.0)	86 (50.6)	61 (70.9)	35.8^a^
>RMB 4500	318 (55.6)	98 (30.8)	534 (53.8)	229 (42.9)	397 (51.4)	184 (46.3)	267 (50.8)	131 (49.1)	198 (53.4)	121 (61.1)	84 (49.4)	42 (50.0)	39.9^a^
Self-reported personality													
Introvert	218 (38.1)	91 (41.7)	403 (40.6)	227 (56.3)	318 (41.2)	191 (60.1)	238 (45.2)	143 (60.1)	168 (45.3)	111 (66.1)	75 (44.1)	54 (72.0)	28.4^a^
Extrovert	354 (61.9)	91 (25.7)	590 (59.4)	270 (45.8)	454 (58.5)	215 (47.4)	288 (54.8)	147 (51.0)	203 (54.7)	112 (55.2)	95 (55.9)	49 (51.6)	44.8^a^
Planned pregnancy													
Yes	456 (79.7)	130 (28.5)	563 (56.7)	237 (42.1)	382 (49.5)	177 (46.3)	274 (52.1)	132 (48.2)	198 (53.4)	104 (52.5)	87 (51.2)	46 (52.9)	44.4^a^
No	116 (20.3)	52 (44.8)	430 (43.3)	260 (60.5)	390 (50.5)	229 (58.7)	252 (47.9)	158 (62.7)	173 (46.6)	119 (68.8)	83 (48.8)	57 (68.7)	13.5^a^
Previous unsuccessful experience (e.g., abortion)													
Yes	177 (30.9)	59 (33.3)	359 (36.2)	213 (59.3)	334 (43.3)	189 (56.6)	212 (40.3)	136 (64.2)	130 (35.0)	75 (57.7)	75 (44.1)	46 (61.3)	17.0^a^
No	395 (69.1)	123 (31.1)	634 (63.8)	284 (44.8)	438 (56.7)	217 (49.5)	314 (59.7)	154 (49.0)	241 (65.0)	148 (61.4)	95 (55.9)	57 (60.0)	58.7^a^
Self-reported health status													
0–59 score	7 (1.2)	5 (71.4)	77 (7.8)	69 (89.6)	88 (11.4)	85 (96.6)	72 (13.7)	63 (87.5)	46 (12.4)	46 (100.0)	26 (15.3)	24 (92.3)	1.9
60–79 score	79 (13.8)	52 (65.8)	238 (24.0)	184 (77.3)	211 (27.3)	149 (70.6)	135 (25.7)	109 (80.7)	96 (25.9)	86 (89.6)	52 (30.6)	44 (84.6)	12.4^a^
80–100 score	486 (85.0)	125 (25.7)	678 (68.3)	244 (36.0)	473 (61.3)	172 (36.4)	319 (60.6)	118 (37.0)	229 (61.7)	91 (39.7)	92 (54.1)	35 (38.0)	13.3^a^
Social support													
12–36 score	5 (0.9)	4 (80.0)	45 (4.5)	36 (80.0)	55 (7.1)	49 (89.1)	34 (6.5)	32 (94.1)	28 (7.5)	27 (96.4)	21 (12.4)	17 (81.0)	1.3
37–60 score	140 (24.5)	77 (55.0)	415 (41.8)	288 (69.4)	391 (50.6)	257 (65.7)	273 (51.9)	178 (65.2)	185 (49.9)	122 (65.9)	93 (54.7)	72 (77.4)	3.3
61–84 score	427 (74.7)	101 (23.7)	533 (53.7)	173 (32.5)	326 (42.2)	100 (30.7)	219 (41.6)	80 (36.5)	158 (42.6)	74 (46.8)	56 (32.9)	14 (25.0)	17.1^a^
Husband's education													
High school or below	194 (33.9)	67 (34.5)	554 (55.8)	305 (55.1)	489 (63.3)	278 (56.9)	323 (61.4)	186 (57.6)	245 (66.0)	150 (61.2)	103 (60.6)	64 (62.1)	22.3^a^
Undergraduate college or above	378 (66.1)	115 (30.4)	439 (44.2)	192 (43.7)	283 (36.7)	128 (45.2)	203 (38.6)	104 (51.2)	136 (34.0)	73 (57.9)	67 (39.4)	39 (58.2)	42.7^a^
Husband's occupation													
White collar	56 (9.8)	16 (28.6)	77 (7.8)	35 (45.5)	60 (7.8)	19 (31.7)	39 (7.4)	17 (43.6)	26 (7.0)	18 (69.2)	14 (8.2)	6 (42.9)	5.3^c^
Blue collar	393 (68.7)	122 (31.0)	600 (60.4)	286 (47.7)	459 (59.5)	245 (53.4)	292 (55.5)	157 (53.8)	192 (51.8)	114 (59.4)	97 (57.1)	61 (62.9)	57.8^a^
Self-employed	76 (13.3)	25 (32.9)	228 (23.0)	127 (55.7)	180 (23.3)	101 (56.1)	135 (25.7)	81 (60.0)	101 (27.2)	61 (60.4)	33 (19.4)	23 (69.7)	12.1^a^
Unemployed	47 (8.2)	19 (40.4)	88 (8.9)	49 (55.7)	73 (9.5)	41 (56.2)	60 (11.4)	35 (58.3)	52 (14.0)	30 (57.7)	26 (15.3)	13 (50.0)	1.1

^a^ *p*  < 0.001.

^b^ *p*  < 0.01.

^c^ *p*  < 0.05.

^d^Linear-by-linear association.

**Table 2:** Logistic regression results of antennal depression among pregnant women at the third trimester in China.

Variables	OR	*p*	95%CI
Age group (ref: 18–24 years)			
25–34	0.788	0.037	0.630, 0.985
35–44	0.673	0.066	0.442, 1.026
Rural (ref: urban)	1.094	0.317	0.917, 1.304
High school or below (ref: undergraduate college or above)	1.110	0.316	0.905, 1.362
Housewife (ref: employed)	1.399	0.012	1.078, 1.817
Average personal monthly income ≤ RMB 4500 (ref: >RMB 4500)	1.031	0.737	0.862, 1.234
Introvert (ref: extrovert)	1.324	0.001	1.119, 1.568
Unplanned pregnancy (ref: yes)	1.303	0.002	1.098, 1.547
Have previous unsuccessful experiences (e.g., abortion) (ref: no)	1.099	0.295	0.921, 1.310
Self-reported health status	0.929	<0.001	0.922, 0.936
Social support	0.948	<0.001	0.940, 0.955
Husband's education is high school or below (ref: undergraduate college or above) Husband's occupation (ref: white collar)	0.919	0.410	0.751, 1.124
Blue collar	1.054	0.751	0.763, 1.455
Self-employed	1.123	0.535	0.779, 1.617
Unemployed	1.160	0.472	0.774, 1.739

## Data Availability

The data are available from the corresponding author upon reasonable request.

## Supporting Information

Supporting Information Supporting Results: Figure S1. Trends in total respondents of sociodemographic characteristics from 2016 to 2021 year. Figure S2. Trends in mild, moderate, and severe ADR from 2016 to 2021 year. Figure S3. Trends in mild, moderate, and severe ADR among 18–43 years old pregnant women.
